# DICOM header editing, between possibilities and regulatory bounds– 40 years of DICOM “standard”

**DOI:** 10.1007/s00259-025-07318-8

**Published:** 2025-05-01

**Authors:** Christian Kuehnel

**Affiliations:** Clinic for Nuclear Medicine, University Hospital Jena, Jena, Germany

## Dear Editor, 

With great interest I read the commentary by Lafontaine et al., “TriDFusion (3DF) image viewer”, and thanks the community for its contributions toward developing forward-thinking solutions [1]. Fortunately, the image data remain unchanged during file transfer; however, the Digital Imaging and Communications in Medicine (DICOM) header still exhibits discrepancies. After 40 years since the establishment of the DICOM standard, formerly known as the ACR/NEMA standard, it is no secret that DICOM is not DICOM when manufacturers and scientists talk about it.

This letter aims to raise awareness for the complexity and consequences of DICOM metadata editing in research and clinical settings, and to suggest approaches for improving traceability and compatibility.

The DICOM standard serves as a cornerstone for medical imaging interoperability. The DICOM data are originally stored in binary format, with both image data and metadata encoded accordingly. The image data consists of raw pixel values, which may be uncompressed or compressed depending on the modality and settings. DICOM stores metadata using a structured approach, where each element consists of a unique tag, a length descriptor, and a corresponding value. While the data is inherently binary, it is often represented in hexadecimal format when viewed with DICOM editors or hex viewers, particularly in the DICOM header, where element tags (e.g., (0020,000D) for the Study Instance UID) follow a standardized format. In the field of nuclear medicine, challenges frequently arise due to the utilisation of the ‘NM’ tag during the subsequent processing of qualitative or quantitative SPECT data.

**Fig. 1:**
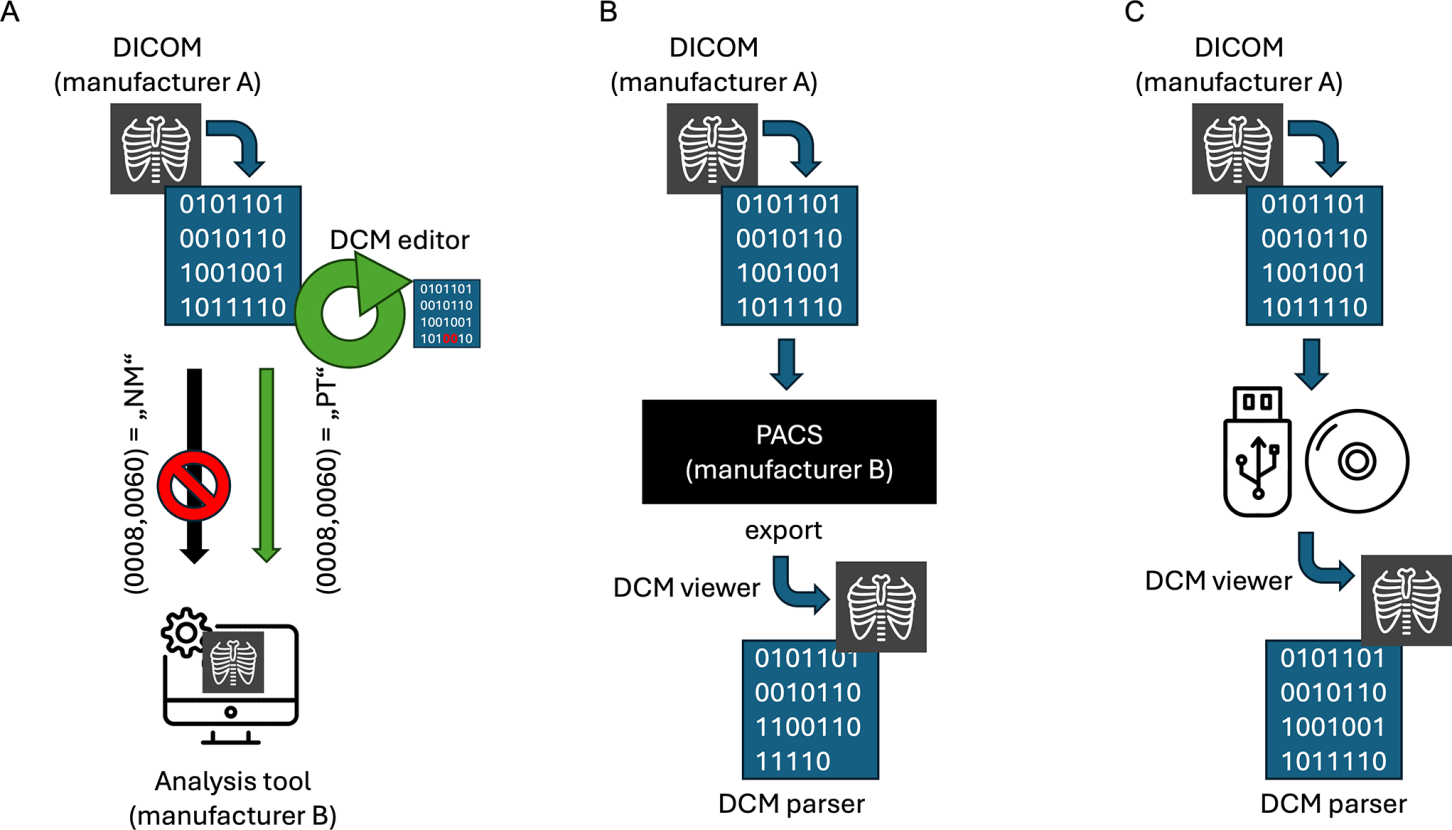
Examples of DICOM data modification. **A**: Intentional modification for readability, e.g. analysis tools or fusion imaging. **B**: Unintentional modification by deleting header tags when switching between different vendors and modalities. **C**: No modification by handling DICOM data from the same system using USB or other data storage devices

Despite established guidelines and consensus-based practices, the editing of DICOM header files remains a complex and sometimes problematic issue. Modifications shown in Fig. 1 may occur either intentionally (driven by research and technical requirements, e.g., modality tag changes), unintentionally (when data are transferred between manufacturer platforms with differing implementations of the DICOM standard, e.g., variations in vector tags for raw quantification permission), or not at all when data are moved via data carriers. It is important to note, however, that the examples mentioned also occur in other constellations. Even when compliance declarations aim to ensure consistency, unintended modifications, such as overwriting or omitting essential metadata fields, frequently occur.

Beyond these technical inconsistencies, research-oriented image processing techniques are increasingly challenging the rigid modality-specific constraints imposed by DICOM and regulatory frameworks. Many advanced post-processing methods (MRI analysis tools such as voxel subtraction) that are currently limited to specific imaging modalities could benefit a wider range of applications if intermodality compatibility were better supported (for example subtracting dynamic PT or SPECT images). For instance, modern sequential fusion approaches with ultrasound (US) are often reserved for certain modality types, such as CT or PT. For SPECT data, this limitation can only be circumvented by changing the tag (0008,0060) from NM to PT [2]. However, the process of DICOM parsing and editing of individual parameters necessitates a certain degree of expertise and can be a time-consuming endeavour. This renders its implementation into a clinical routine somewhat challenging.

From a research perspective, modifying DICOM headers offers new opportunities to evaluate emerging methodologies within established from other modality frameworks. Yet, any alteration of metadata conflicts with ethical, regulatory and legal considerations, particularly when it affects patient safety, clinical interpretation, or the intended regulatory use of medical imaging devices. Furthermore, modifying DICOM tags to circumvent modality-specific restrictions could potentially extend medical devices beyond their conformity assessment scope, with unpredictable legal implications.

To address these challenges, manufacturers should consider intermodality compatibility in certification processes, ensuring that image processing tools can be utilized across different imaging systems without requiring unauthorized modifications. A more flexible approach within regulatory frameworks could foster innovation while maintaining patient safety and compliance. Encouraging collaboration between researchers, industry, and regulatory bodies is crucial to defining a pathway that balances technological progress with ethical and legal accountability. To this end, initiatives by professional bodies such as the EANM Physics Committee could help establish guidelines on permissible metadata edits in research contexts and their appropriate documentation.

In addition to existing integrity checks such as DICOM Digital Signatures, a blockchain-inspired approach could offer a new way to enhance transparency and traceability in image data management. By providing a tamper-proof record of any changes to metadata, such a system would help ensure data integrity, especially in research environments where header modifications are common [3]. This, in turn, would enhance reproducibility, accountability, and compliance with ethical and regulatory standards. The integration of blockchain technology into DICOM infrastructures has the potential to harmonise technological innovation with the rigorous demands of clinical practice and regulatory oversight.

Looking ahead, it is to be hoped that we will not require another 40 years to establish a universally accepted and transparent standard for DICOM metadata handling—one that balances the needs of research, clinical practice, and regulatory compliance in a coherent and future-oriented manner.

## Data Availability

N/A.
